# Editorial: Immune cell dynamics and biomarkers in cardiac surgery-induced systemic inflammatory response

**DOI:** 10.3389/fcvm.2026.1788870

**Published:** 2026-02-24

**Authors:** Jia Tan, Bingyang Ji, Yongfeng Shao, Weixun Duan, Wataru Takayama, Lei Du

**Affiliations:** 1 Department of Anesthesiology, West China Hospital, Sichuan University, Chengdu, Sichuan, China; 2 State Key Laboratory of Cardiovascular Medicine, Department of Cardiopulmonary Bypass, Fuwai Hospital, National Center for Cardiovascular Disease, Chinese Academy of Medical Sciences & Peking Union Medical College, Beijing, China; 3 Department of Cardiovascular Surgery, Jiangsu Province Hospital, The First Affiliated Hospital of Nanjing Medical University, Nanjin, China; 4 Department of Cardiovascular Surgery, Xijing Hospital, The Air Force Medical University, Xi'an, China; 5 Department of Anesthesiology, Kawasaki Saiwai Hospital, Kawasaki, Japan

**Keywords:** cardiac surgery, cardiopulmonary bypass, immune cell, immunotherapy, systemic inflammatory response

Systemic inflammatory response (SIR) triggered by cardiac surgery under cardiopulmonary bypass (CPB) remains an unresolved challenge leading to multi-organ dysfunction, infection, and increased mortality (1, 2). To alleviate SIR, numerous studies have tried to removal pro-inflammatory cytokines and neutrophils. Unfortunately, no strategies have yet translated into effective preventive or therapeutic treatment, because of the complex interplay of immune activation (3). This Research Topic was launched to address two interrelated questions: how does immune status shape patient outcomes, and can immune modulation effectively improve prognosis? This topic comprises eight articles, trying to explore potential inflammatory and immune biomarkers and their relationship with immune status and clinical outcomes, as well as evaluating the effectiveness of immunotherapy and perioperative immunomodulation.

In this topic, Jia et al. found that early low postoperative serum prealbumin was independently associated with pulmonary complications and new-onset atrial fibrillation in patients undergoing off-pump coronary artery bypass grafting. Yu et al., Aierken et al., and Li and Cai independently demonstrated that the systemic immune-inflammation index (SII) and several ratios derived from leukocyte counts and lipid parameters are associated with mortality, stroke progression, and in-hospital death. Maisat et al. demonstrated marked postoperative remodeling of gene expression in pediatric patients undergoing CPB. Downregulation of myo5c correlated consistently with organ dysfunction scores, interleukin-6 (IL-6) levels, and markers of neutrophil and platelet activation, which may be associated with neutrophil extracellular trap formation. These identified indices derived from routine complete blood counts and biochemical tests are easily accessible, repeatable, and low-cost, making them attractive for clinical risk stratification.

The interventional studies in this topic have explored perioperative immune modulation and organ protection. In patients undergoing cardiac surgery, Huang et al. found that bilateral transversus thoracis plane block improved by dexmedetomidine significantly reduced perioperative stress hormone levels and shortened the duration of mechanical ventilation and ICU stay. In an experimental venoarterial extracorporeal membrane oxygenation (VA-ECMO) model, Chen et al. found that early left heart decompression improved oxygenation and hemodynamics, mitigated pulmonary edema, and reduced inflammatory cytokine levels in lung tissue, thereby strengthening the link between mechanical stress, inflammation, and organ injury. Jiefei et al. demonstrated that intraoperative HA380 blood adsorption for infective endocarditis was associated with shorter hospital stay and more favorable coagulation-inflammation profiles, suggesting that non-specific cytokine removal might improve aspects of the inflammation without impacting main clinical endpoints.

Overall, the studies collected in this Research Topic convey two key messages. First, the association between immune status and clinical outcome is beyond doubt, yet existing markers still fall short of enabling precise prediction and targeted intervention. Second, cytokine networks are highly complex, with short half-lives and strong feedback regulation, preventive strategies centered only on cytokines and non-specific anti-inflammatory approaches appear insufficient to substantially improve outcomes. These observations point towards a necessary shift in strategy: from targeting cytokines to targeting cells.

At present, there are two main cell-based strategies for treating SIR. Selective Cytopheretic Device (SCD), developed in the United States, was initially designed for patients undergoing CPB, but its relatively low blood flow and processing efficiency led to a shift toward use in dialysis patients. SCD is integrated into the continuous renal replacement therapy (CRRT) circuit and operated under low shear stress and low ionized calcium conditions. It takes advantage of the enhanced adhesiveness of activated leukocytes, which preferentially adhere to the fiber membrane surface. In patients requiring renal replacement therapy, SCD treatment has been shown to improve survival, but it carries a risk of inducing hypocalcemia (4). Another strategy is the Selective Activated Leukocyte Depletion (SALD) device developed in China. This device uses an ultrafine fiber adsorption membrane grafted with RGD peptides (Arg–Gly–Asp), which can selectively bind to specific peptide structures on activated leukocytes and thereby achieve efficient removal of activated leukocytes. In patients undergoing cardiac surgery with CPB, SALD has been shown to reduce activated leukocyte burden (5). Compared with SCD, SALD is simple to use and efficient to remove the activated leukocytes. Now, both of them are being conducted in accordance with Good Clinical Practice (GCP), and we expect they would bring good news for lower organs dysfunction and lower mortality.

Building on these insights, perioperative immune phenotyping may be used to identify patients most likely to benefit from immunotherapy, followed by the selective adsorption or modulation of specific immune cell subsets to reprogram their functional states rather than simply lowering mediator levels. We believe that only when immune monitoring, risk stratification, and therapeutic strategies form a closed loop will it be possible to achieve a genuine breakthrough in the management of SIR. The studies presented in this Research Topic lay an important foundation for this goal and provide clear direction for future clinical and translational research on immune cells in the perioperative setting.
